# Cell death pathway induced by resveratrol-bovine serum albumin nanoparticles in a human ovarian cell line

**DOI:** 10.3892/ol.2015.2851

**Published:** 2015-01-07

**Authors:** LIYUAN GUO, YAN PENG, YULIAN LI, JINGPING YAO, GUANGMEI ZHANG, JIE CHEN, JING WANG, LIHUA SUI

**Affiliations:** 1 Department of Obstetrics and Gynecology, Third Affiliated Hospital, Harbin Medical University, Harbin 150081, P.R. China; 2 Department of Health, First Affiliated Hospital, Heilongjiang University of Chinese Medicine, Harbin 150040, P.R. China; 3 Department of Health, First Affiliated Hospital, Harbin Medical University, Harbin 150001, P.R. China

**Keywords:** resveratrol-bovine serum albumin nanoparticles, apoptosis, programmed cell death, caspase-independent

## Abstract

Resveratrol-bovine serum albumin nanoparticles (RES-BSANP) exhibit chemotherapeutic properties, which trigger apoptosis. The aim of the present study was to investigate the caspase-independent cell death pathway induced by RES-BSANP in human ovarian cancer SKOV3 cells and to analyze its mechanism. Morphological changes were observed by apoptotic body/cell nucleus DNA staining using inverted and fluorescence microscopy. The cell death pathway was determined by phosphatidylserine translocation. Western blot analysis was conducted to detect the activation of apoptosis-inducing factor (AIF), cytochrome *c* (Cyto *c*) and B-cell lymphoma 2-associated X protein (Bax). Apoptotic body and nuclear condensation and fragmentation were observed simultaneously following treatment with RES-BSANP. RES-BSANP induced apoptosis in a dose-dependent manner in the human ovarian cancer SKOV3 cells. The translocation of AIF from the mitochondria to the cytoplasm occurred earlier than that of Cyto *c*. In addition, Bax binding to the mitochondria was required for the release of AIF and Cyto *c* from the mitochondria. The AIF apoptosis pathway may present an alternative caspase-dependent apoptosis pathway in human ovarian cell death induced by RES-BSANP. Elucidation of this pathway may be critical for the treatment of cancer using high doses of RES-BSANP.

## Introduction

Resveratrol (RES) exhibits anti-inflammatory and anti-oxidant effects (1,2) and is a potential chemopreventive agent, which is able to inhibit various stages of carcinogenesis, including the initiation, promotion and progression of tumors (3). RES has been found to induce apoptotic cell death in various cancer cell lines and experimental tumor models (4–7).

Previously, it has been reported that high doses of RES (3 g/kg/day) cause moderate liver toxicity (8). However, the absorption of RES was shown to be poor due to its water insoluble properties (9). Furthermore, the insolubility of RES has been demonstrated to hamper the *in vitro* and *in vivo* biological induction of RES activity (10). Research in nanomedicine has not only become a frontier movement, but is also a revolutionary drug delivery field. Bovine serum albumin (BSA) has been used as a vehicle used for diagnostic and therapeutic agents (11,12), as it is non-toxic, safe, economical and exhibits good biocompatibility and no immunogenicity. Previous studies have explored BSA as a drug carrier (13,14).

RES-BSA nanoparticles (RES-BSANP) are synthesized by protein desolvation chemical crosslinking. Previous studies have demonstrated the antiproliferative effects of RES on the growth of human SKOV3 cell lines in *in vivo* and *in vitro* studies (15–17). The prepared RES-BSANP exhibited an altered distribution. In addition, it was demonstrated that RES-BSANP-treated tumors exhibited a similar apoptotic index to RES control tumors. The cells in the therapeutic groups exhibited apoptotic and necrotic characteristics. The mechanism resulting in these properties may be the release of cytochrome *c* (Cyto *c*) and the upregulation of the dynamic expression of caspase-9 and -3, indicating that the mitochondrial apoptotic pathway was activated (16,17).

Programmed cell death (PCD) is a specific mechanism that initiates cell death. Caspases are involved in PCD. PCD may be divided into two types, caspase-dependent and caspase-independent PCD, according to the involvement of caspase in PCD. Caspase-dependent PCD presents typical apoptosis. Caspase-independent PCD includes autophagy, paraptosis, mitotic catastrophe, apoptosis-like PCD and necrosis-like PCD.

RES-BSANP has been found to inhibit various stages of tumor growth, however, the molecular mechanism of its anticancer activity remains unclear, particularly in ovarian cancer. The aim of the present study was to elucidate the molecular events that occur during RES-BSANP-induced apoptotic cell death in human ovarian SKOV3 cells.

## Materials and methods

### Reagents

The human ovarian cancer SKOV3 cell line was obtained from the Tumor Research Institute of Harbin Medical University (Harbin Medical University, Harbin, China). RES was purchased from Xian Huacui Biology Co., Ltd. (Xian, China), (purity, ≥99.9%) and dissolved in dimethyl sulfoxide (DMSO) as a stock solution of 100 mmol/l. RES was further diluted in Dulbecco’s modified Eagle’s medium (DMEM) with 10% fetal bovine serum (FBS) to the appropriate final concentrations. RES-BSANP was prepared at the Life Science Laboratory of Northeast Forestry University (Harbin, China).

The general caspase inhibitor, Z-VAD-FMK [Z-Val-Ala-Asp(OMe)-CH_2_F], and caspase-9 [Z-LEHD-FMK, Z-Leu-Glu(OMe)-His-Asp(OMe)-CH_2_F] were obtained from Calbiochem (La Jolla, CA, USA). Stock solutions of the caspase inhibitors (10 mmol/l each) were prepared in DMSO and diluted in DMEM with 10% FBS to a final concentration of 100 μmol/l. Polyclonal rabbit anti-rat antibodies against apoptosis-inducing factor (AIF), Cyto *c* and B-cell lymphoma 2 (Bcl-2)-associated X protein (Bax) were obtained from Beijing Zhongshan Golden Bridge Biotechnology Co., Ltd. (Beijing, China). DMEM, penicillin (1 mg/ml) and streptomycin (1 mg/ml) were obtained from Invitrogen Life Technologies (Carlsbad, CA, USA) and FBS was purchased from HyClone Laboratories, Inc.,(Logan, UT, USA). The 2.5% trypsin/EDTA solution was purchased from Invitrogen Life Technologies and diluted to 0.5% for trypsinizing attached cells.

### Cell culture

The ovarian cancer SKOV3 cell line was cultured in RPMI 1640 (Gibco-BRL, Carlsbad, CA, USA) supplemented with 10% heat-inactivated fetal calf serum (FCS; Gibco-BRL) in a humidified incubator at 37°C, with an atmosphere of 5% CO_2_. A total of 2.5 g/l trypsin and 0.2 g/l EDTA were used for subculture.

### Morphological study

To evaluate apoptotic cell death, the cells were seeded at a density of 5×10^5^ cells/ml in 15-mm diameter wells and incubated for 12 h until the cells had adhered to the bottom of the wells. DNA ladder formation was examined an hour following the start of treatment with the compounds. For morphological examination of the apoptotic changes, the cells were stained with Hoechst 33342 (5 μg/ml; Invitrogen Life Technologies) at 37°C for 30 min, washed twice with phosphate-buffered saline, pipetted drop-wise onto a glass slide, and examined by fluorescence microscopy using an Olympus microscope (Olympus Corporation, Tokyo, Japan) equipped with an epi-illuminator and appropriate filters.

### DNA fragmentation

DNA fragmentation was conducted as previously described (18). Following various treatments, the cells were collected and lysed with lysis buffer [0.5% Triton X-100, 5 mmol/l Tris Buffer (pH 7.4), 20 mmol/l EDTA]. RNA was removed by incubation with RNase A (0.8 mg/ml) at 37°C for 30 min. DNA was extracted by phenol/chloroform and precipitated with 1/10 volume of 3 mol/l sodium acetate (pH 5.2) along with 20μl of 100% ethanol. DNA pellets obtained by centrifugation at 10,000 × g for 20 min at 4°C, were dried and re-suspended in 25 ml 1xTAE (40 mmol/l Tris-acetate and 1 mmol/l EDTA). Samples were then separated on 2% agarose gels and the DNA ladder was detected by incubation of the gels with ethidium bromide (1 g/ml) for 20 min followed by de-staining with distilled H_2_O.

### Inhibition of apoptosis by the pan-caspase inhibitor

The tripeptide pan-caspase inhibitor, Z-VAD-FMK was added 12 h prior to treatment with RES-BANP. The optimal concentration of the inhibitor was determined from a dose-response curve using the extent of cell death. The inhibition of apoptosis by Z-VAD-FMK was evaluated by investigating the inhibition of nucleosomal DNA fragmentation, which was observed as DNA ladder formation.

### Western blot analysis

Western blot analysis was performed to detect the protein expression of AIF, Cyto *c* and Bax. Cell lysate was prepared by lysis buffer protein extraction [40 mmol/l Tris-Cl (pH 8.0), 120 mmol/l NaCl and 0.1% NP40] supplemented with protease inhibitors. Proteins were separated by SDS-PAGE and transferred to nitrocellulose membranes (Bio-Rad, Hercules, CA, USA). The membranes were blocked with 5% skimmed milk in Tris-buffered saline and incubated with the appropriate primary antibodies for 1 h at room temperature. The blots were developed with peroxidase-conjugated secondary antibody, and the proteins were visualized with an enhanced chemiluminescence kit (GE Healthcare, Piscataway, NJ, USA) according to the manufacturer’s instructions.

### Statistical analysis

Results are expressed as the mean ± standard deviation. For multiple comparisons, results were analyzed by one-way analysis of variance. The least significant difference was analyzed by the Bonferroni correction test to identify significant differences between the individual cell groups. P<0.05 was considered to indicate a statistically significant difference. All statistical analyses were performed using SPSS software, version 11.5 (SPSS, Inc., Chicago, IL, USA).

## Results

### Cell detection via microscopic observations

Following observation under an inverted phase contrast microscope (IX73-F22FL/PH, Olympus Corp., Tokyo, Japan) and a fluorescence microscope (CKX41, Olympus Corp.), numerous cells were found in the culture flask of the control group. The cells had adhered to the flask wall. The level of cell latency and refraction was high and the proliferation rate of the tumor cells was also high. Treatment with 10, 50 and 80 μmol/l RES-BSANP for 2, 4, 6, 8, 12 and 24 h resulted in morphological cell changes, as observed by apoptotic body/cell nucleus DNA staining. The size of the cell bodies in the experimental groups treated with 50 μmol/l RES-BSANP for 8 h were reduced significantly and had become round in shape. The latency and refractivity of the cells was weakened, with vacuoles appearing in the cytoplasm. As the concentration of RES-BSANP and the exposure time increased, the cells began to shrink and rupture. The morphology of the SKOV3 cells became irregular, with surrounding debris, and the culture medium became turbid. A large number of cells collapsed, floated and died. The difference between apoptosis and necrosis induced by 50 μmol/l RES-BSANP is shown in Table I.

### DNA fragmentation

**Figure 1:**
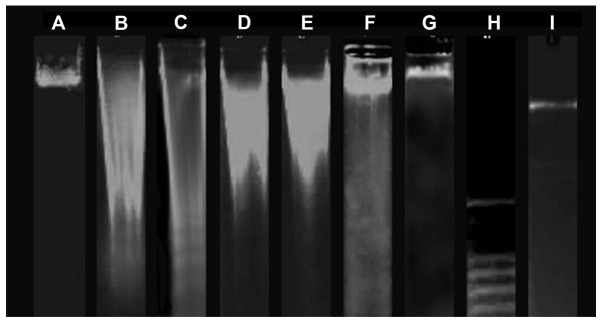
DNA Fragmentation. A, control; B, 20 μmol/l RES-BSNAP; C, 50 μmol/l RES-BSNAP; D, 80 μmol/l RES-BSNAP; E, 100 μmol/l RES-BSNAP; F, 50 μmol/l Z-VAD-FMK and RES-BSANP; G, 50 μmol/l Z-VAD-FMK; H, λDNA/*Hin*dIII; I, 100-base pair DNA ladder. RES-BSANP, resveratrol-bovine serum albumin nanoparticles.

To further verify the apoptotic response, DNA fragmentation was examined. DNA ladder formation was observed in SKOV3 cells treated with 50 μmol/l RES and 20 μmol/l RES-BSANP for 4 h. Treatment duration was then extended to 6 h. Large scales of DNA ladder were detected, in contrast to the 50 μmol/l Z-VAD-FMK control group. These results indicated that the marked suppression of cell growth by RES and RES-BSANP was attributable to apoptotic cell death. Gel electrophoresis exhibited DNA ladders and smears in the high-dose RES-BSANP groups (80 and 100 μmol/l RES-BSANP) and exhibited only a smear in the Z-VAD-FMK control group (Fig. 1).

### Western blot analysis of AIF, Cyto c and Bax expression in SKOV3 cells

**Figure 2:**
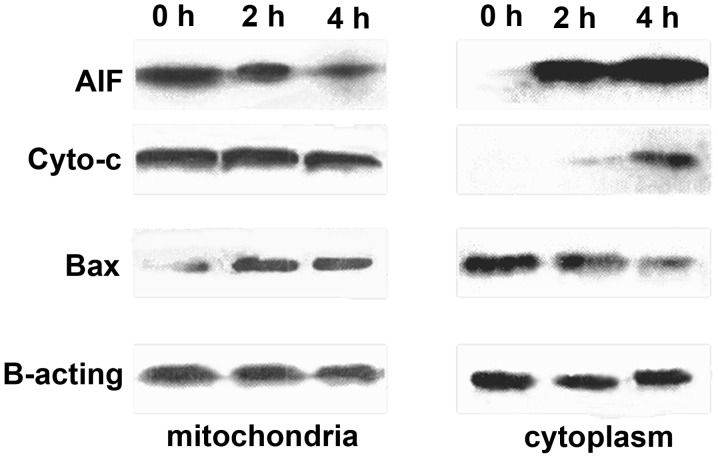
Western blot analysis of AIF, Cyto *c* and Bax protein expression following treatment with 50 μmol/l resveratrol-bovine serum albumin nanoparticles for 0, 2 and 4 h. AIF, apoptosis-inducing factor; Cyto *c*, cytochrome *c*; Bax, B-cell lymphoma 2-associated protein X.

Induction of apoptosis by RES-BSANP may involve the translocation of Cyto *c* and AIF. This translocation was investigated by biochemically fractionating different subcellular compartments and quantifying the expression of Cyto *c* and AIF by western blot analysis. Cells treated with 80 μmol/l RES-BSANP for 2 h showed a spatiotemporal release of AIF from the mitochondria into the nucleus (Fig. 2). Similarly, it appeared that Cyto *c* was also released from the mitochondria, however, notably, this was not accompanied by a concomitant cytoplasmic increase at 4 h of treatment. The translocation of AIF from mitochondria to cytoplasm occurred earlier than that of Cyto *c.* These results indicated that RES-BSANP-elicited cell death may not occur via a classical Cyto *c* mitochondria cytosol translocation mechanism but rather, a caspase-independent mechanism of cell death via the nucleus directed shuttling of AIF and Cyto *c*.

Activation of Bax and its mitochondrial translocation are the key regulatory events during induction of mitochondrial membrane depolarization. Treatment of macrophages with macrophage inflammatory protein supernatant resulted in mitochondrial translocation of Bax as early as 2 h following treatment. Reduction of AIF and Cyto *c*, resulted in increased mitochondrial Bax. The binding of Bax to the mitochondria was required for the release of AIF and Cyto *c* from mitochondria.

## Discussion

Previous studies have identified two primary forms of cell death, namely apoptosis and necrosis. Apoptosis was initially considered the primary physiological and programmed form of cell death. The apoptotic proteins involved with the caspase-dependent pathway include Cyto *c*, caspase-9 and caspase-3 (16,17). Cell death via this pathway is associated with the activation of caspases. However, it is now widely recognized that PCD may also occur in the absence of caspase activation (18–22). The existence of non-caspase PCD pathways has been found to be associated with caspase-independent elimination, including the use of mitochondrial protein AIF (19,20,23,24). The current study revealed that the caspase-independent pathway, mediated by AIF, may be involved in the necrotic PCD pathway, particularly following treatment with high doses of RES-BSANP in cell culture.

Caspase activation is regarded as a vital factor in RES-BSANP-induced cell death in *in vitro* and *in vivo* studies. However, previous studies have indicated that RES-induced cell death in human ovarian cancer cells is caspase-independent (17,18); the treatment of these cells with RES-BSANP resulted in the release of Cyto *c* and the activation of caspase-3. Apoptotic cell death is induced by RES-BSANP and appears to be caspase-independent, as caspase inhibitors fail to attenuate induced cell death by RES treatment. It has previously been reported that AIF mediates cell death via a caspase-independent pathway (25). Mitochondrial AIF translocates to the nucleus as a result of cell death stimuli and thus initiates nuclear condensation (26,27). Once the nucleus condenses, this leads to large-scale chromatin fragmentation, followed by cell death. Consistent with these observations, the current study demonstrated translocation of AIF from the mitochondria to the nucleus following RES-BSANP treatment of the SKOV3 cells. AIF translocation and nuclear condensation were detectable within 24 h of RES-BSANP treatment. Whilst AIF protein is considered to be a potent factor in caspase-independent apoptosis, the mechanism by which AIF causes DNA ladder formation remains unclear.

It has been reported that AIF protein release is localized around the cell nuclei and is partly translocated into these nuclei following treatment with the apoptogenic dolichylmonoposphate in SKOV3 cells. Caspase-3 and -8 inhibitors prevent not only DNA fragmentation, but also AIF migration and chromatin condensation (28). In the current study, the pan-caspase inhibitor prevented induced apoptotic cell death following RES-BSANP treatment, indicating that AIF protein release, rather than caspase release, may be pivotal in DNA ladder formation. These results are consistent with previous studies of RES-BSANP-induced apoptosis.

To the best of our knowledge, this is the first study to investigate the caspase-independent signaling mechanism of RES-BSANP-induced apoptosis in SKOV3 cells. Further studies are required to elucidate the precise signaling pathways involved in the RES-BSANP-induced apoptotic death of ovarian cancer SKOV3 cells. Notably, RES-BSANP exhibited a potent effect on the SKOV3 cells, which indicates that polyphenol compounds may also be a candidate for a chemo-preventive and chemotherapeutic agent, as the doses of the compounds used can be considerably lower than the chemotherapy drugs commonly in use currently to exhibit the same activity as RES.

It has been reported that a member of the Bcl-family, Bax, is associated with the release of AIF from the mitochondria (29,30), and the release of Ca^2+^ from the endoplasmic reticulum appears to be important in this process (31). Results of previous studies indicate that the release of AIF, with a decrease in membrane potential and modulation of the expression of Bax may be partly responsible for RES-BSANP-induced apoptosis in SKOV3 cells. The polyphenol compounds have been shown to affect activation of caspase-independent cell death pathways and thus exhibit an anti-cancer activity. Recently, the understanding of how RES induces cell death in human ovarian cancers has markedly improved. RES usually inhibits signaling via the mitogen-activated protein kinase and phosphatidylinositol 3-kinase/AKT pathways (32–36). Consistently, RES has been found to suppress the activity of the downstream transcription factors AP-1 and nuclear factor-κB (RelA/p65) (35–38). Genes that are considered to be transcriptionally affected by RES with an impact on apoptosis include cyclins, cyclin-dependent kinases, caspases, p53, p21 (Cip1/WAF1), p300, NF-κB, Bcl-2, Bax and inhibitors of apoptosis (39,40). Consequently, further studies investigating RES-BSANP’s role in cell death, excluding the caspase-dependent and caspase-independent pathways, are required. A great deal remains be determined with regard to the mechanism of cell necrosis or cell death by treatment with RES-BSANP.

Taken together, these results indicate that the main signal transduction pathway of RES-BSANP induced apoptosis in SKOV3 cells is mediated by the activation of caspase. Concurrently, additional intracellular signaling pathways may also be involved. Further research is required to clarify the association between the early response signal and the apoptotic signal. Thus, RES-BSANP, which is a constituent of an anti-tumor compound, may be a potentially effective candidate for chemoprevention.

## Figures and Tables

**Table I:** Effects of resveratrol-bovine serum albumin nanoparticles (80 μmol/l) on apoptotic body/cell nucleus DNA staining.

	Duration of treatment, h					
	
Cell count	2	4	6	8	12	24
Apoptotic bodies	22±0.21	25±2.6	47±3.51	56±0.8	59±2.6	63±6.4
Necrosis	0.44±0.5	64±0.6^a^	87±5.6^a^	102±4.5^b^	130±4.7^a^	178±1.3^b^

a P<0.05,

b P<0.01, compared with apoptotic bodies.

Data are presented as the mean ± standard deviation.
